# Supplementary material for: Neural mediation of greed personality trait on economic risk-taking
Source: eLife. 2019 Apr 29;8:e45093. doi: 10.7554/eLife.45093 (PMC6506209; doi:10.7554/eLife.45093)
Supplement: Supplementary file 1. [file elife-45093-supp1.docx]

**Supplementary file 1 -Tables for statistical results and model parameters.**

**Table S1. Descriptive statistics and model fitting parameters in fMRI and behavioral replication studies.**

|  | **fMRI study (n=25)**  **[mean (s.d)]** | **Behavioral replication study (n=49)**  **[mean (s.d)]** |
| --- | --- | --- |
| Greed personality trait (GPT) | 22.280 (3.669) | 22.690 (4.736) |
| Impulsivity personality trait (IPT) | 34.967 (10.042) | 33.707 (9.804) |
| Percentage of risky mixed choices | 0.422 (0.204) | 0.387 (0.185) |
| Percentage of risky gain choices | 0.495 (0.204) | 0.471 (0.211) |
| Percentage of risky loss choices | 0.494 (0.218) | 0.485 (0.209) |
| Behavioral loss aversion (*λ*) | 1.561 (0.724) | 1.711 (0.687) |
| Risk attitude (*α*) | 1.033 (0.201) | 1.037 (0.182) |
| Choice consistency (τ) | 1.029 (0.936) | 0.970 (1.079) |

**Table S2. Contribution of impulsivity and greed personality trait scores in behavioral and neural regression analyses for the fMRI dataset.**

| **Dependent Variable** | **Regressors** | | | |
| --- | --- | --- | --- | --- |
|  | **Impulsivity** | | **Greed** | |
|  | **Coefficient (β)** | ***T* (*p*)** | **Coefficient (β)** | ***T* (*p*)** |
| Percentage of risky mixed choices | -0.006 | -1.315(0.202) | 0.029 | 2.296 (0.032) |
| Percentage of risky gain choices | -0.003 | -0.562(0.580) | 0.016 | 1.138(0.267) |
| Percentage of risky loss choices | 0.005 | 0.961(0.347) | -0.0001 | -0.010(0.992) |
| Behavioral loss aversion (*λ*) | 0.017 | 1.074(0.294) | -0.112 | -2.557(0.018) |
| Behavioral risk attitude (*α*) | -0.005 | -1.109(0.280) | 0.004 | 0.290(0.775) |
| Neural loss aversion (τ) | 0.002 | 1.704(0.103) | -0.008 | -2.359(0.028) |
