# Supplementary material for: Neural mediation of greed personality trait on economic risk-taking
Source: eLife. 2019 Apr 29;8:e45093. doi: 10.7554/eLife.45093 (PMC6506209; doi:10.7554/eLife.45093)
Supplement: Supplementary file 2. [file elife-45093-supp2.docx]

**Supplementary file 2 - The discriminant and convergent validities of Dispositional Greed Scale (DGS) in the datasets.**

|  | **Samples** | | | |
| --- | --- | --- | --- | --- |
|  | **N = 25**  **(fMRI)** | **N = 49**  **(Behavioral)** | **N = 76**  **(Pilot Questionnaire)** | **N = 150**  **(All subjects combined)** |
| **Dispositional Greed Scale (DGS) Items** | **Factor loading** | **Factor loading** | **Factor loading** | **Factor loading** |
| 1. I always want more. | 0.849 | 0.786 | 0.754 | 0.755 |
| 2. Actually, I'm kind of greedy. | 0.512 | 0.741 | 0.704 | 0.681 |
| 3. One can never have too much money. | 0.189 | 0.501 | 0.598 | 0.526 |
| 4. As soon as I have acquired something. I start to think about the next thing I want. | 0.339 | 0.632 | 0.509 | 0.533 |
| 5. It doesn't matter how much I have. I'm never completely satisfied. | 0.795 | 0.711 | 0.826 | 0.776 |
| 6. My life motto is "more is better". | 0.909 | 0.747 | 0.719 | 0.750 |
| 7. I can't imagine having too many things. | 0.055 | 0.747 | 0.609 | 0.596 |
| **Eigenvalue** | 2.595 | 3.439 | 3.250 | 3.115 |
| **Explained variance** | 0.371 | 0.491 | 0.464 | 0.445 |
| **Cronbach's alpha** | 0.611 | 0.819 | 0.798 | 0.783 |
|  |  |  |  |  |
| **Correlation with impulsiveness and behavioral measures** | **r (p-value)** | **r (p-value)** | **r (p-value)** | **r (p-value)** |
| Impulsiveness | 0.543 (0.005) | 0.117 (0.424) | 0.272 (0.018) | 0.254 (0.002) |
| Risk attitude | -0.077 (0.715) | -0.087 (0.553) | - | - |
| Risk attitude after controlling IPT | 0.062 (0.775) | -0.089 (0.547) | - | - |
| Loss aversion | -0.439 (0.028) | -0.332 (0.020) | - | - |
| Loss aversion after controlling IPT | -0.479 (0.018) | -0.325 (0.024) | - | - |

***Note.*** IPT: impulsivity personality trait scores.
