# Supplementary material for: Neural mediation of greed personality trait on economic risk-taking
Source: eLife. 2019 Apr 29;8:e45093. doi: 10.7554/eLife.45093 (PMC6506209; doi:10.7554/eLife.45093)
Supplement: Supplementary file 3. [file elife-45093-supp3.docx]

**Supplementary file 3 - Tables of brain activations correlating with potential gains and losses.**

**Table S1. Brain regions responding to potential gains.**

| **Location** | **MNI Coordinates**  **X Y Z** | | | **Z value** | **Cluster size** |
| --- | --- | --- | --- | --- | --- |
| **Heightened activity as gains increased** | | | | | |
| Bilateral medial frontal cortex  Bilateral orbitofrontal cortex | -4  -6 | 52  48 | 0  -10 | 4.72  4.34 | 542 |
| Left posterior cingulate | -8 | -62 | 20 | 4.50 | 177 |
| Left middle temporal cortex | -48 | -6 | -22 | 4.19 | 177 |
| Right middle temporal cortex | 48 | 0 | -18 | 4.29 | 144 |
| Left parietal cortex | -56 | -24 | 18 | 4.38 | 89 |
| Right parietal cortex | 46 | 0 | 50 | 4.28 | 34 |
| **Decreased activation as gains increased** | | | | | |
| Left middle frontal cortex  Left insula | -40  -34 | 10  20 | 32  -2 | 5.89  5.20 | 1248 |
| Right insula | 32 | 28 | 2 | 4.54 | 157 |
| Left supplementary motor area | -6 | 14 | 48 | 5.24 | 845 |
| Bilateral occipital cortex  Left parietal cortex | 40  -26 | -68  -66 | -14  44 | 5.58  5.25 | 7518 |
| Right inferior frontal cortex | 48 | 16 | 28 | 4.83 | 404 |

*Note.* Results were FDR cluster corrected (*z* = 3.1, *P*_FDR_ <. 05). FDR, False Discovery

Rate.

**Table S2. Brain areas responding to potential loss.**

| **Location** | **MNI Coordinates**  **X Y Z** | | | **Z value** | **Cluster size** |
| --- | --- | --- | --- | --- | --- |
| **Decreased activation as the magnitudes of losses increased** | | | | | |
| Bilateral medial frontal cortex | -4 | 34 | 40 | 5.23 | 418 |
| Left striatum | -10 | 12 | 0 | 4.96 | 133 |
| Right striatum | 12 | 10 | 6 | 5.06 | 136 |
| Left insula | -36 | 18 | 0 | 4.97 | 95 |
| Right insula | 30 | 26 | 0 | 4.10 | 75 |
| Left inferior parietal cortex | -48 | 38 | 16 | 5.17 | 638 |
| Right inferior parietal cortex | 44 | -52 | 46 | 5.20 | 1279 |
| Left superior medial cortex | -4 | 34 | 40 | 5.23 | 428 |
| Left inferior frontal cortex | -42 | 46 | 4 | 4.76 | 638 |
| Right middle frontal cortex | 46 | 36 | 22 | 5.49 | 1178 |
| Left middle temporal cortex | -56 | -40 | -12 | 4.99 | 428 |
| Right middle temporal cortex | 60 | -48 | -8 | 5.06 | 354 |

*Note.* Results were FDR cluster corrected (z = 3.1, *P*_FDR_ <. 05).
